# The Effect of Inhaled Beta-2 Agonists on Heart Rate in Patients With Asthma: Sensor-Based Observational Study

**DOI:** 10.2196/56848

**Published:** 2024-12-11

**Authors:** Rishi Jayant Khusial, Jacob K Sont, Omar S Usmani, Matteo Bonini, Kian Fan Chung, Stephen James Fowler, Persijn J Honkoop

**Affiliations:** 1 Department of Biomedical Data Sciences Leiden University Medical Center Leiden Netherlands; 2 National Heart and Lung Institute Imperial College London & Royal Brompton and Harefield NHS Trust London United Kingdom; 3 Department of Public Health and Infectious Diseases Sapienza University Rome Italy; 4 NIHR Manchester Biomedical Research Centre (BRC) University of Manchester, and Manchester University NHS Foundation Trust – Wythenshawe Hospital Manchester United Kingdom

**Keywords:** asthma, mHealth, side effects, beta-2 agonists, inhaler medication, heart rate, sensor, observational study, asthma management, cardiac cells, monitoring, Fitbit, inhaler

## Abstract

**Background:**

Beta-2 agonists play an important role in the management of asthma. Inhaled long-acting beta-2 agonists (LABAs) and short-acting beta-2 agonists (SABAs) cause bronchodilation by stimulating adrenoceptors. These receptors are also present in cardiac cells and, as a side effect, could also be stimulated by inhaled beta-2 agonists.

**Objective:**

This study aims to assess the effect of beta-2 agonists on heart rate (HR).

**Methods:**

The data were retrieved from an observational study, the myAirCoach Quantification Campaign. Beta-2 agonist use was registered by self-reported monthly questionnaires and by smart inhalers. HR was monitored continuously with the Fitbit Charge HR tracker (Fitbit Inc). Patients (aged 18 years and older) were recruited if they had uncontrolled asthma and used inhalation medication. Our primary outcome was the difference in HR between LABA and non-LABA users. Secondary outcomes were the difference in HR on days SABAs were used compared to days SABAs were not used and an assessment of the timing of inhaler use during the day.

**Results:**

Patients using LABA did not have a clinically relevant higher HR (average 0.8 beats per minute difference) during the day. Around the moment of SABA inhalation itself, the HR does increase steeply, and it takes 138 minutes before it returns to the normal range*.*

**Conclusions:**

This study indicates that LABAs do not have a clinically relevant effect on HR. SABAs are instead associated with a short-term HR increase.

**Trial Registration:**

ClinicalTrials.gov NCT02774772; https://clinicaltrials.gov/study/NCT02774772

## Introduction

Asthma is a highly prevalent inflammatory disease, affecting over 300 million people worldwide [1]. Asthma symptoms, including coughing, wheezing, and shortness of breath, are the result of several different pathological processes, resulting in the constriction of smooth muscle cells in the airways, which causes a reversible airflow obstruction [2]. To locally target the inflammatory component of asthma, inhaled corticosteroids (ICS) were developed, and they are still the mainstay of asthma therapy [3]. Beta-2 agonists were developed to specifically target airway constriction and thereby relieve symptoms. Since asthma is a chronic disease, it is usually necessary for patients to take their medication regularly to keep their asthma in control.

Short-acting beta-2 agonists (SABAs) are widely used in case of a sudden worsening of symptoms [3]. They used to be the preferred choice as reliever medication, although with the 2019 Global Initiative for Asthma (GINA) guideline update, they are now the second choice, and SABA-only treatment in adults is discouraged because it increases the risk of asthma-related death [4]. Currently, for the first medication step in asthma treatment, GINA recommends to use of ICS-formoterol on an as-needed basis to reduce symptoms [3]. In the following step, the use of ICS-formoterol is preferred to be taken daily. Formoterol is a form of long-acting beta-2 agonist (LABA). The bronchodilator effect of LABAs is noticeable after 5-20 minutes and lasts for approximately 12 hours [5-7]. For most brands, patients are advised to use their LABA inhaler twice daily. SABAs last for 3-6 hours and their effect is most noticeable after a few minutes.

Inhaled beta-2 agonists are sympathomimetics that mimic the working mechanism of noradrenaline or adrenaline. They activate beta-2 adrenergic receptors on the lining of the bronchial muscles, resulting in the relaxation of these muscle cells and dilation of the airways. Also, inhaled beta-2 agonists inhibit mast cell mediator release and plasma exudation, and they could also reduce the activation of sensory nerves [8]. However, beta-2 adrenoceptors are not only present in the lungs but also in other parts of the body, including the heart.

Conversely, oral beta blockers are commonly prescribed for multiple cardiac diseases and work the opposite way. Beta-blockers prevent noradrenaline or adrenaline from connecting to the adrenoreceptors, resulting in a lowering of the heart rate (HR) and, as a potential side effect, constriction of the airways. On average, beta-blockers lower HR by about 8-21 beats per minute (bpm) [9].

Therefore, since inhaled beta-2 agonists prescribed for asthma activate beta-2 receptors, it is possible that they might stimulate the sympathetic system. In previous literature, it has already been described that inhaled beta-2 agonists are associated with systemic side effects, including myocardial infarction [10] and cardiac arrest [11] by beta-2 receptor stimulation of cardiac cells [12]. It is to be expected that the systemic effects with regard to increasing HR by inhaled beta-2 agonists are less than the systemic effects with regard to decreasing HR by beta-blockers since the latter are taken orally. In an experimental setting, it was shown that LABAs can be safely used [13]. However, the actual real-life effect of LABA and SABA uses on HR is largely unknown.

An elevated HR is a serious side effect, which warrants further investigation. Increased HR is a strong independent risk factor for the development of cardiomyopathy, coronary artery disease, fatal myocardial infarction, sudden death, cardiovascular mortality, and total mortality [12].

In this observational study, we assessed pulse rate as an approximation of HR continuously for 1 year with sensor-based wristwatches, while also registering physical activity levels and medication adherence, and the exact timing of beta-2 agonist inhalation with the use of smart inhalers. These data gives us the opportunity to assess the real-life effects of beta-2 agonists on HR.

Therefore, in this study, we aim to assess the relationship between beta-2 agonists (LABAs and SABAs) and HR.

## Methods

### Subjects

For this study, the dataset acquired for the multinational European Horizon2020 myAirCoach Quantification Campaign was used [14]. The original study aimed to develop predictive models for asthma control based on data obtained by home monitoring, mobile health sensors, and environmental databases. To this purpose, patients were given various technological devices to monitor physiological parameters. Patients were eligible for this study if they were aged 18 years and older, had a clinical diagnosis of asthma (as diagnosed by a general practitioner or pulmonologist), and had an affinity for technology (as indicated by the patient himself). Additional inclusion criteria were the occurrence of a severe exacerbation in the year prior to the study or an Asthma Control Questionnaire score>1.5 at the last control visit with their regular asthma health care provider (general practitioner, pulmonologist, or nurse), indicating uncontrolled asthma [15]. Furthermore, patients are required to be in asthma treatment steps 2-5 [3]. Patients were excluded if they suffered from severe comorbidity (including diagnosis with a life expectancy of less than 1 year) and had insufficient language proficiency (Dutch or English for the research center in the Netherlands or English for the research centers located in the United Kingdom).

### Ethical Considerations

The study was approved by the Medical Ethics Committee of the Leiden University Medical Centre and Imperial College London (reference number NL54495.058.15) and the NHS Health Research Authority (REC reference 15/NW/0845; IRAS project ID 185603). All participants provided written informed consent upon entering the study. Data were anonymized to safeguard privacy and confidentiality.

### Design

The myAirCoach Quantification Campaign was an observational study performed in the Netherlands (Leiden) and the United Kingdom (London and Manchester) with 12 months of follow-up. The participants received several electronic devices and an iPod Touch (Apple Inc). On the iPod, apps required for the technological devices were preinstalled. More details on the myAirCoach Quantification Campaign can be found in the previously published study protocol [14].

#### Fitbit

The continuous monitoring of the heart rate was assessed with the Fitbit Charge HR (Fitbit Inc). The Fitbit Charge HR is a “wearable” commonly known as a fitness tracker. Every single minute of the HR data is stored as a separate measurement. The Fitbit Charge HR also measures the number of steps taken, stairs walked, calories burned, and minutes slept. It was advised to only wear the Fitbit at night if patients were comfortable wearing the device during sleep.

#### Inhaler Use

Participants reported their medication use at the start of the trial. LABA use was documented through questionnaires. In a weekly questionnaire, participants were asked if their medication had changed. The exact timing of the use of medication was recorded using the smart inhaler by Adherium (currently available as “Hailie”; Multimedia Appendix 1). This is an inhaler add-on that records when the inhaler is used. The patient kept using their regular inhaler (including canister) prescribed by their asthma health care provider. The smart inhaler itself is a small extra “casing” for the regular inhalers. Patients were able to put their own inhaler inside the smart inhaler and independently replace the canister once empty. The research team did not make any medication adjustments. Separate smart inhalers were available for SABA, ICS, and ICS/LABA inhalers. When the term LABA is used in this paper, both LABA and ICS/LABA inhalers are meant since LABAs can be prescribed as a single combination inhaler with ICS or as a stand-alone LABA inhaler to be used in combination with an ICS inhaler. Data on stand-alone ICS use were also gathered but not further assessed in this study.

### Outcomes

#### Primary Outcome

Our primary outcome was the difference in HR throughout the day between patients prescribed with LABA and patients not prescribed with LABA. For this purpose, we divided patients into an LABA group and a non-LABA group. In a weekly questionnaire, patients reported if they used LABA. When a participant added or removed LABAs to or from their treatment, the patient also switched to the corresponding group for that part of the follow-up. Fitbit provides data as the mean HR per minute. Maximum follow-up was 365 days, and if a patient wore his or her Fitbit for fewer than a number of minutes in a week (7×60×60 minutes), the participant was removed from the analysis. For this comparison, these results were recalculated into the mean HR per hour, and subsequently mean scores per group per hour were compared. There is no known minimally clinically important difference in HR increase or reduction for the general population [16]. However, in cardiological literature, a meta-regression analysis in people with heart failure showed that the relative risk for death decreased by 18% for every 5-bpm sustained reduction in HR with beta-blocker treatment [9]. This indicates that a sustained increase of 5 bpm could be considered clinically relevant, especially for patients with asthma and cardiovascular comorbidity.

#### Secondary Outcomes

Next to LABAs, we also assessed the effect of SABAs on HR. Since SABAs are used on an “as needed” basis and they are short-acting, we looked at their effect on HR around the moment of inhalation. It is described that SABAs are regularly used directly before physical exercise, which in itself also greatly influences HR [17]. Therefore, we used Fitbit data to analyze the number of steps taken in relation to SABA use.

### Smart Inhaler

Additionally, we analyzed the patterns in HR in the hours prior to and after LABA and SABA inhalation in patients with a smart inhaler. The smart inhaler was attached to the inhaler the patient used normally and was switched by the patients themselves when they used a new canister of inhalation medication.

### Statistical Analyses

We analyzed HR throughout the day for patients using LABA compared to patients not using LABA. Adherence to wearing the Fitbit device differed significantly between participants. To avoid overreliance on the data of individuals wearing the device more often, a mean HR per hour of the day was calculated for each individual before calculating the mean HR per LABA use group. Using the smart inhaler data, we examined the HR in the minutes around the inhalation more closely.

For SABAs, patients only use them as reliever medication, next to their controller medication. Therefore, we were able to use participants as their own controls, to have the most accurate comparison. We assessed the mean HR in 180 minutes before and 600 minutes after SABA use. We compared these to the exact same minutes (of the day) on 100 days (50 days before and 50 days after the SABA inhalation) the participant did not use SABAs (control days). For the control days, we looked at the HR at the exact same time of the day as the time SABAs were used. For example, if a patient uses SABAs at 11 AM, HR is assessed in the hours before and after 11 AM. HR data of a total of 100 non-SABA days in the hours before and after 11 AM were used as control data. Mean HR per control minute was calculated. We chose to look at (a maximum of) 100 control days to account for personal differences in activities people do during the year that could influence HR (eg, someone could be more active in a specific period in the year). By using this individual comparison, we accounted for individual HR differences, as well as for the effect of the circadian rhythm on HR. To ensure we were not measuring the effect of physical exercise on HR, we assessed the mean number of steps and compared these to the mean + 2 SDs amount during the same time period for the 100 non–SABA-use days. We only assessed the effect of SABAs on HR if the mean number of steps in the period surrounding the actuation was less than the mean + 2 SDs in the 100-day control period.

Data were analyzed with STATA version 14 (StataCorp).

## Results

### Overview

For this study, data from a total of 94 patients were available. The baseline characteristics of these patients are reported in Table 1, divided between patients who used LABAs and patients who did not use LABAs. This distinction between the 2 groups is based on their medication use at the beginning of the study. Not all patients in the myAirCoach Quantification Campaign recorded their HR. Out of 94 patients, 85 participants partly filled in the baseline questionnaire. All patients were included in the study since they filled in subsequent questionnaires and recorded Fitbit data.

**Table 1:** Baseline characteristics.

		Patients using LABAs^a^ (n=65^b^)	Patients not using LABAs (n=17^b^)	Total (N=85^b^)	*P* value
**Sex, n (%)**					
	Female	50 (77)	12 (71)	65 (76)	.59
**Age (years), mean (SD)**		43.8 (12.1)	37.5 (11.8)	42.81 (12.2)	.06
**BMI, mean (SD)**		26.48 (4.6)	26.16(5.2)	26.07 (4.9)	.83
**Smokers, n (%)**					
	Currently	3 (5)	1 (6)	4 (5)	N/A^c^
	Former	9 (14)	3 (18)	12 (15)	N/A
	Never	53 (81)	13 (76)	66 (80)	N/A
**Asthma-related hospitalization previous year, n**		14	1	15	.39
**Age of diagnosis (years), mean (SD)**		15.7 (14.4)	16.7 (16.5)	16.9 (15.4)	.81
**FEV1** ^d^ **, mean (SD)**		2.49 (0.98)	2.31 (0.92)	2.48 (0.95)	.86
**ACD** ^e^ **, mean (SD)**		1.48 (1.03)	1.20 (1.24)	1.39 (1.08)	.30
**AQLQ** ^f^ **, mean (SD)**		4.67 (1.16)	5.36 (1.14)	4.82 (1.18)	.02

^a^LABA: long-acting beta-2 agonist.

^b^Not all patients filled out the entire baseline questionnaire (including questions relating to their LABA use). They did, however, record heart rate and filled out subsequent questionnaires and were therefore included in analysis, from the moment medication status was known.

^c^N/A: entry is not applicable.

^d^FEV1: forced expiratory volume in the first second.

^e^ACD: Asthma Control Diary.

^f^AQLQ: Asthma Quality of Life Questionnaire.

### Self-Reported LABA Use

At baseline, 25 patients did not use LABAs, compared to 69 patients who reported they used LABAs. In total, 13 patients were removed from analyses because they wore Fitbit less than a week (mean 2870 minutes). During the study, patients changed 13 times between groups: patients changed 7 times from the LABA group to the non-LABA group and 6 times from the non-LABA group to the LABA group. Some of the patients (n=3) changed more than once between groups. Participants were asked to continuously wear their Fitbit, but since it is an electronic device, it had to be charged every few days, and no specific instructions were given when patients should charge their device. A total of 18.7% of the data were recorded during the night (between 12 AM and 6 AM), resulting in approximately 3 million nighttime measurements.

**Figure 1:**
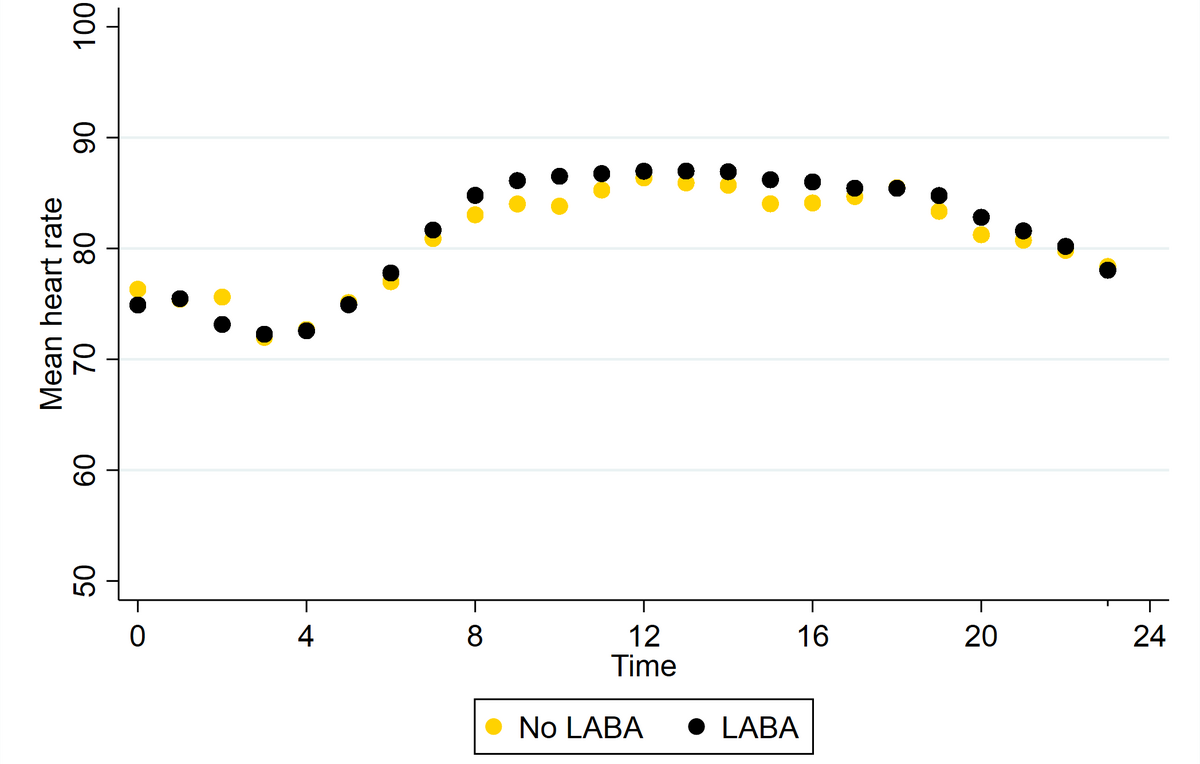
Self-reported LABA use and heart rate throughout the day. The time on the x-axis is clock hours. Heart rate during follow-up with and without LABA use follows a normal circadian rhythm. The mean heart rate per patient per hour was calculated first. Next, the mean score is calculated by LABA follow-up. This is done so the number of measurements per patient would not influence results. LABA: long-acting beta-2 agonist.

Overall, the HR difference between LABA and non-LABA users was minimal (mean difference 0.8, 95% CI 0.8-0.8 bpm). This difference was most pronounced in the morning, as shown in Figure 1.

### Smart Inhaler Data

**Figure 2:**
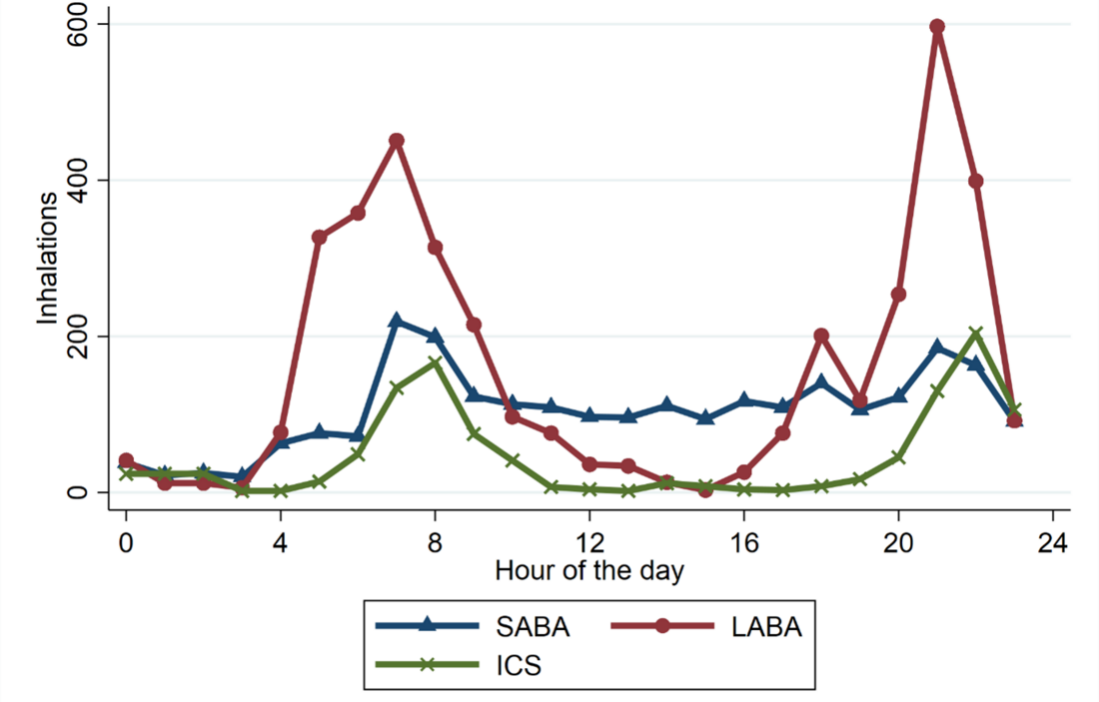
Inhalations. The number of inhalations is recorded with the smart inhaler. Patients used smart inhalers for their inhaled medication in their treatment as usual. The number of inhalations can therefore not be compared to each other. Patients used their maintenance medication (ICS or ICS/LABA) twice daily and their rescue inhalation during the day equally, except for a minor peak between 8 AM and 9 AM. ICS: inhaled corticosteroid; LABA: long-acting beta-2 agonist; SABA: short-acting beta-2 agonist.

A subset of 24 patients was provided with smart inhalers. They recorded a total of 7388 inhalations. In Figure 2, inhaler use during the day is plotted. The figure shows that maintenance therapy (ICS or ICS/LABA) is generally used to conform prescription twice daily, once in the morning and once in the evening. SABA use was consistent during the day with a peak around 8 AM.

**Figure 3:**
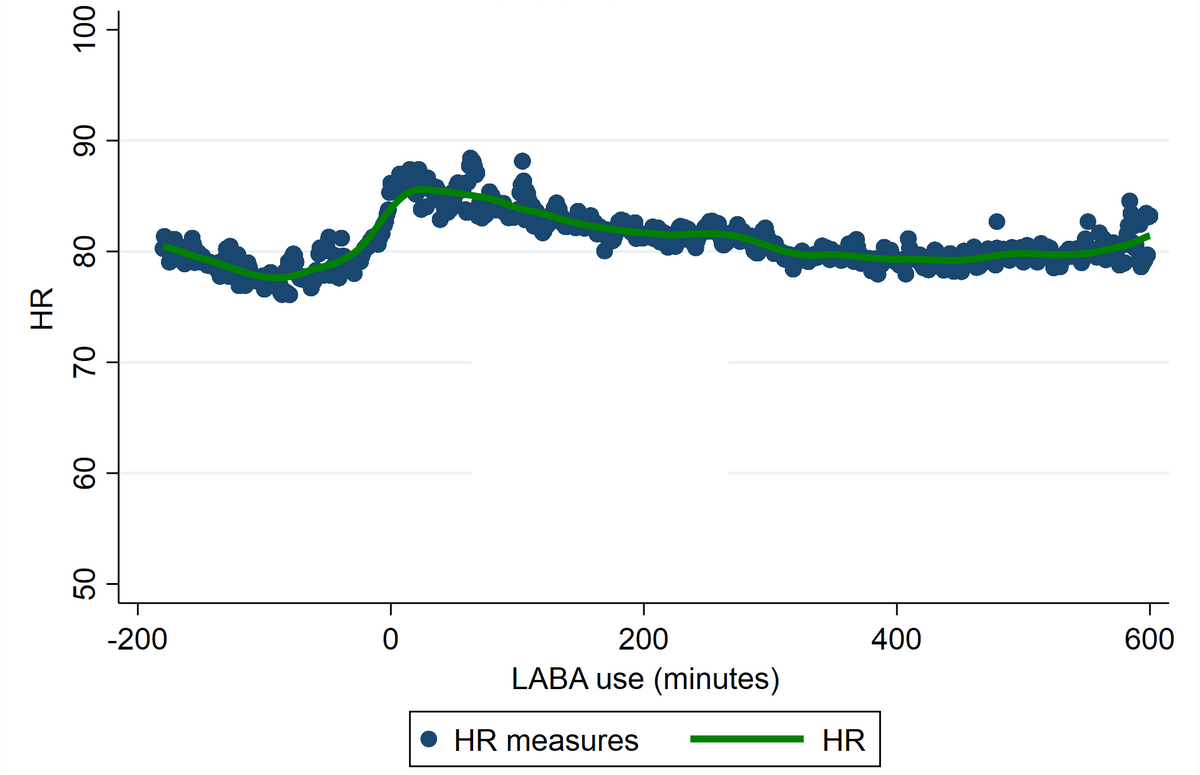
HR around LABA use as measured with the smart inhaler. HR: heart rate; LABA: long-acting beta-2 agonist.

Figure 3 shows a sharp rise in HR around the time of LABA inhalation, which gradually decreases over 5 hours after inhalation. Minute 0 is the minute the LABA inhalation is taken. The green line is a smoothed trend line of these measurements.

**Figure 4:**
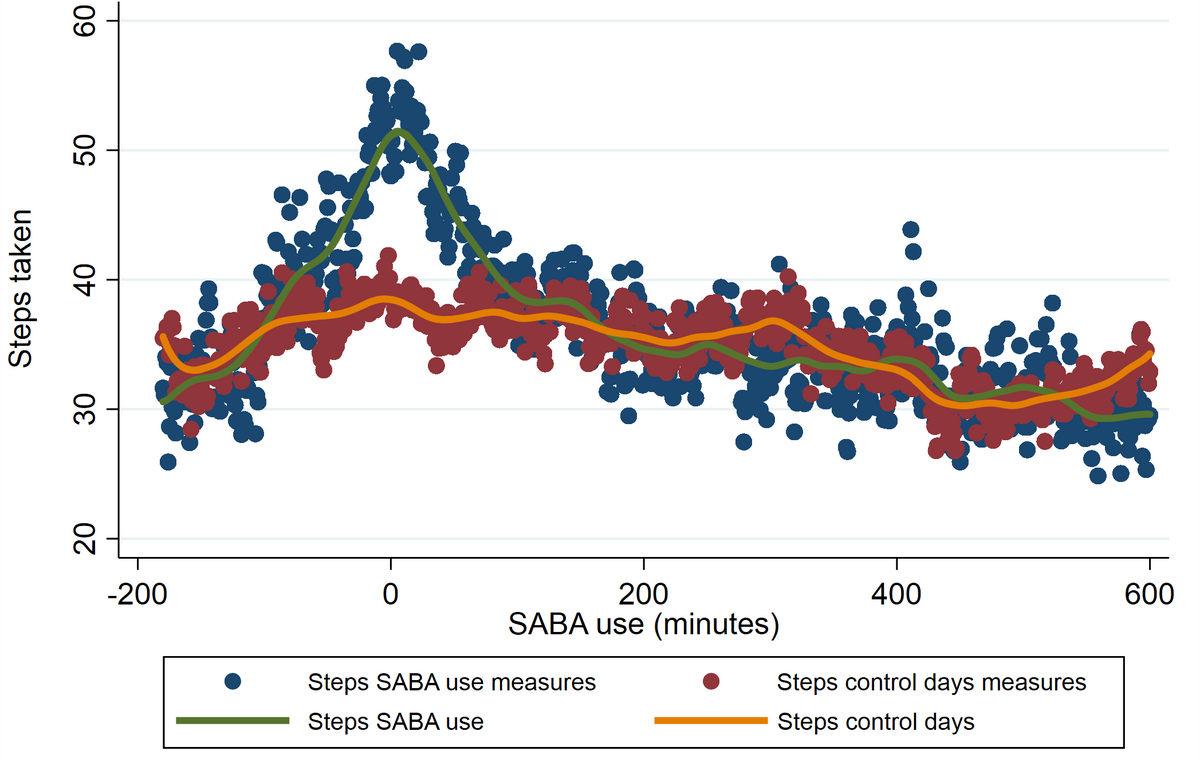
Steps taken around SABA inhalation. SABA: short-acting beta-2 agonist.

In Figure 4, patients had an increased number of steps surrounding the SABA inhalation compared to days they did not use SABAs. Most steps are taken around the time of inhalation. The green line is a smoothed trend line of the steps on the days SABA inhalation is taken and the yellow line is the smoothed trend line of the steps on the control days.

**Figure 5:**
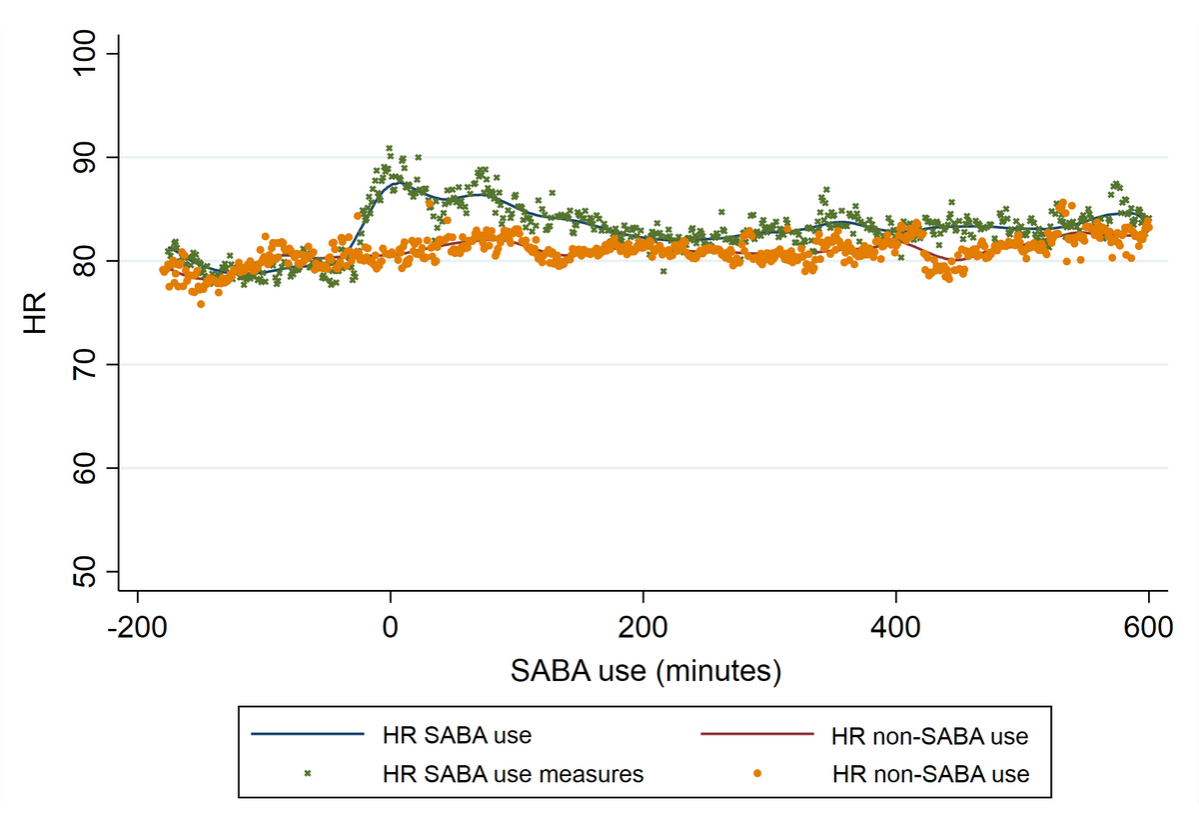
SABA inhalation registered with the smart inhaler and HR. HR: heart rate; SABA: short-acting beta-2 agonist.

In Figure 5, minute 0 is the minute the SABA inhalation is taken. The green crosses in the graph represent individual measurements (from 3 hours before until 10 hours after inhalation) around a SABA inhalation. The blue line is a smoothed trend line of these measurements. The yellow dots depict the mean heart rate of the same patients, at the same time of SABA inhalation (on inhalation days), and on days they did not use SABAs. Adjustments for steps taken, recorded by the Fitbit (Figure 4), were made by excluding measurements if the number of steps taken in the inhalation period (1 hour before and 1 hour after inhalation) exceeds the mean + 2 SD number of steps taken by the same person on the days he did not use SABA. A peak is noted around inhalation and a higher HR is sustained for approximately 2 hours after inhalation.

### SABA Data

SABAs with a smart inhaler were used by 19 different patients in 2510 cases. As shown in Figure 4, more steps are taken around inhalation. The steps start to increase approximately 30 minutes before the actuation and the increase lasts approximately 90 minutes after actuation. This clear increase in steps assessed in the entire population indicates that SABA inhalation is often used during exercise. In Figure 5, it is shown that the HR after inhalation is higher than the HR on non–SABA-use days. This figure also shows the initial sharp rise in HR around the inhalation moment itself. The difference in HR between SABA and non-SABA inhalation days exceeds 5 bpm for the first time at 17 minutes before inhalation. At the time of inhalation, HR was 9 bpm higher than the HR on non-SABA days. HR difference was widest at 9 minutes after inhalation (10 bpm) after which HR difference slowly decreased. HR difference then heavily fluctuated in the minute-by-minute comparisons and after 138 minutes no further clinically relevant HR difference≥5 bpm was observed.

## Discussion

### Principal Findings

This study shows that the use of LABAs is not associated with a clinically relevant HR elevation. In an overall comparison, patients using LABAs had a maximum HR of 3 bpm higher in the morning (at 10 AM) than patients not using LABAs. The sheer amount of data collected makes every small difference between groups statistically significant. Therefore, even though the HR difference found in this study is statistically significant, it does not seem clinically relevant.

SABA use is associated with a short-term increase in HR, which peaks 9 minutes after inhalation and then steadily decreases until it normalizes 138 minutes after inhalation.

### Previous Literature

Cardiac side effects of LABAs were also assessed in several other studies, but the follow-up duration of these studies was limited due to technological restrictions and patients were not assessed in a real-life setting [18-20]. For example, Braden et al [21] measured the HR with Holter-ECG connected to the thorax of the patient. Overall, these studies showed between 5 and 15 bpm increase in HR. This could be attributed due to the sharp, but short rise in HR following the inhalation.

Salpeter et al [12] performed a meta-analysis and found that compared with a placebo, single-dose inhaled beta-2 agonists significantly increased HR (by 9 bpm). These studies looked at the short-term effect of beta-2 inhalation on HR. They gave no information on the duration of this increase. Our data suggest this increase after SABA use could be sustained for 138 minutes. In long-term studies assessed in the meta-analysis; they showed a nonsignificant trend in major cardiovascular events [12].

### Strengths and Weaknesses

This study shows a new method of using daily technology for the analysis of health care–related research questions in a real-life setting. While many studies are performed in a semicontrolled setting, real-world situations are often different. In a real-life setting, patients often use SABAs as needed next to their daily LABA inhaler. Compared to previous studies, with the use of smart inhalers, we were able to specifically look at the added effect of SABAs on HR when patients used LABA [18-31]. Even if real-life measurements might be less accurate than data collected in an experimental setting, the amount of data that could be collected for a longer period makes the results useful. Especially when patients can “wear” the measurement devices (wearables), it is not a major burden for them to participate in studies.

As an important strength of this study, we had extensive HR data of participants, since they were wearing Fitbit for approximately a year, and every minute data were recorded. In total, we had 7388 inhalations recorded with the smart inhaler (2510 SABA inhalations, 3823 ICS/LABA inhalations, and 1055 ICS inhalations) and over 15 million data points with HR. The mean number of HR measurements per patient was 188,296 (SD 137,479; 130 days’ worth of minutes) and the median was 157,295 measurements (IQR 61,670-293,204). Our study analyzed the effects of both LABAs and SABAs. This gives a more complete overview of the relationship between beta-2 agonists and cardiac activity. Finally, we had data during the day and night, and these were obtained in a real-life setting.

A limitation is the number of patients used in the analyses and it is also important to note that we did not specifically look at LABAs as a rescue medication. Even though Figure 2 shows 2 peak moments for LABA use (morning and evening), some inhalers (known as “maintenance and reliever therapy”) include an LABAs that could also be used on an “as needed” basis. Since patients in our study were not able to record if they used LABAs as rescue medication, we were not able to account for this.

Another important limitation is the inability to completely adjust findings for physical activity. Even though we tried correcting for the increase in steps taken, other forms of physical activity increase are not picked up by Fitbit since physical activity could also be increased without an increase in steps, for example, weightlifting. An increase in HR could therefore partly be attributed to exercise. The inhalation maneuver itself could also be related to the HR increase since this sharp rise is also seen with the LABA smart inhaler data.

Since LABAs were used in the maintenance therapy in most patients, the control group was relatively small. In 84% of the cases, patients took their morning LABA medication every day, and in less than 7% of the cases, patients forgot their morning medication for more than 2 consecutive days. Remarkably, a comparable HR increase around inhalation is seen in LABAs as with SABA inhalations. As shown in Figure 2, LABAs are used most commonly twice a day (morning and evening) at variable times and HR fluctuates normally during the day. Therefore, it is difficult to interpret if the HR increase noted in Figure 3 is a sustained effect of LABA use or if HR would have increased on its own due to circadian rhythm effects. Since Figure 1 shows no difference in overall HR, changes in circadian rhythm are probably at least partially responsible.

In previous studies, the accuracy of wearables is under debate. While some papers show that in general wearables can accurately predict HR [32-36], this may not hold true for higher HR zones [37]. The Fitbit is not registered as a medical device and may underestimate individual measurements [38] and in a direct comparison with other wearables, the Fitbit showed lower accuracy [39] and higher bias [34]. However, with each new version, the accuracy is improving. Also, Fitbit was used in all patients. There is no reason to assume a systematic difference between the groups (LABAs vs non-LABAs and SABAs vs control minutes) would occur. Therefore, even if Fitbit is erroneous, it will not have affected our results overtly.

Another issue during the study was that some patients reported difficulties with the smart inhaler. They reported that the smart inhaler lost connection to the iPod or the device was in another way malfunctioning so not all inhalations are recorded. Next, both the actuation of the inhaler and the HR were rounded off to the minute, this could result in a theoretical 1-minute difference between inhalation and HR registration. Combined with a possible delay in data transmission, this could result in a small shift in the time data. Especially around inhalation, this could shift the moment HR starts to increase and the moment of highest HR.

### Interpretation and Future Studies

In the LABA and SABA registration by the smart inhaler (Figures 3 and 5), a sharp rise surrounding the inhalation itself is observed, which could indicate that the inhalation maneuver itself could affect HR. Also, in the SABA analyses, we reported that HR started to increase before inhalation took place, and shortness of breath, resulting in SABA use, could also increase HR. Potentially, some of the previous literature that reported an HR increase, with shorter follow-up may have included this effect in their outcomes. We also noticed that after a SABA inhalation, the HR is higher than the days patients did not use SABAs. This increase is sustained for approximately 2 hours, after which the difference between SABAs and non-SABAs is minimal. This difference can be caused by the systemic effects of SABAs on HR. However, other potential causes for HR elevation are physical activities that do not increase steps taken and asthma-related shortness of breath with associated anxiety. With the current data, it is not possible to know what the real-life situation was, and future studies are needed, both in experimental and real-life settings to assess the direct effect of SABAs on HR.

In previous editions of the international GINA guidelines (until 2012), there used to be a preference for short-acting muscarinic agonists over SABAs in patients with cardiac comorbidity. Due to the slow onset of the bronchodilator effect of short-acting muscarinic agonists over SABAs, this has changed. In the current GINA guidelines, the preferred reliever medication track recommends the use of low-dose ICS-formoterol as needed as reliever medication. SABAs are an alternative reliever medication [40]. This study shows that beta-2 agonist use has no sustained clinically relevant effect on HR, which is reassuring. However, there is a temporary increase in HR, which warrants further study.

### Conclusions

Patients with asthma using inhaled LABAs do not experience a clinically relevant persistent increase in HR. SABAs are associated with a short-term elevation in HR.

## Appendix

Multimedia Appendix 1 Smartinhaler.

## Abbreviations

bpm: beats per minute
GINA: Global Initiative for Asthma
HR: heart rate
ICS: inhaled corticosteroid
LABA: long-acting beta-2 agonist
SABA: short-acting beta-2 agonist
