# Supplementary figures and images for: The Effect of Inhaled Beta-2 Agonists on Heart Rate in Patients With Asthma: Sensor-Based Observational Study
Source: JMIR Cardio. 2024 Dec 11;8:e56848. doi: 10.2196/56848 (PMC11669870; doi:10.2196/56848)

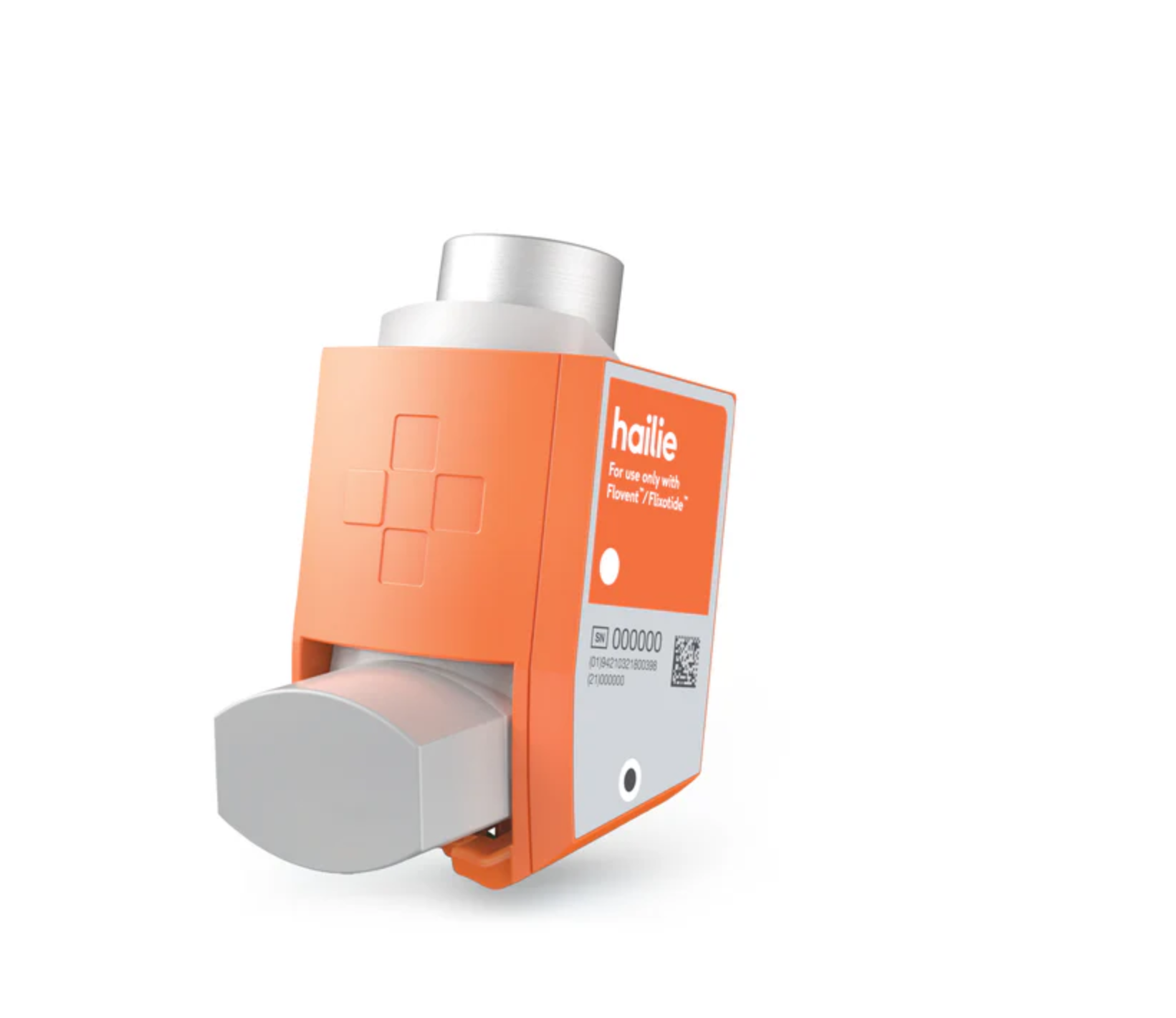

Supplement: Multimedia Appendix 1 [file cardio_v8i1e56848_app1.png]
